# Atlanta Society of Medicine

**Published:** 1896-02

**Authors:** W. L. Champion

**Affiliations:** Secretary


### Society Reports.

### THE ATLANTA SOCIETY OF MEDICINE.

### Regular Meeting, January 7, 1896.

The society was called to order by the president, Dr. C. D. Hurt. The minutes of the previous meeting were read and approved. The reports for 1895 of the secretary and treasurer were read and approved.

At the request of the secretary and treasurer, Dr. Love moved that the president appoint a committee of two to audit the reports of the secretary and treasurer and to report at the next meeting. The motion was carried and the president appointed, on this committee, Drs. Love and Dunbar Roy.

The next in order of business was the election of officers for the year 1896, which resulted as follows: President, R. R. Kime; vice president, W. A. Crow; treasurer, L. B. Grandy; secretary, W. L. Champion; corresponding secretary, Dunbar Roy.

Dr. Duncan moved that the society instruct the treasurer to send a check for $50.00 to the Y. M. C. A, as a slight donation from the society for the privileges of the hall. Motion carried.

### DISCUSSION OF TYPHOID FEVER.

The president appointed Dr. Grandy to discuss the symptoms and diagnosis of typhoid fever. He stated as follows:

"When I came here to-night, I did not expect to be called upon to discuss this, but I would say that the diagnosis of typhoid fever in ordinary cases ought not to present any special difficulties, as long as we have anything like the characteristic train of symptoms of headache, hæmorrhage from the nose, difference in the morning and evening temperature, pain in the abdomen and, when after the fifth or sixth day, the characteristic rash begins to show itself. But the trouble will come from those that do not present these typical features. The chief trouble, as far as my experience has been, is knowing where to distinguish between typhoid cases and those of the ragged edge of remittent fever. Some authors say that typhoid and malarial fevers cannot exist in the same person. However, there are cases which begin with a most strikng resemblance to remittent fever, but resist the treatment for remittent fever and develop into typhoid fever. It is these cases which will trouble us. Some writers class all these as true typhoid fever. Dr. Osler says there is no such thing as typhomalarial fever. Whether it is this or not, there certainly does exist a fever in which the most striking features of typhoid are absent, specially the variations in the morning and evening temperature, pain in the abdomen, and, possibly, the rash on the skin. Those cases would hardly seem to be classed as typical typhoid, yet at any rate, as remittent fever, they do not respond as they should to large doses of quinine. My experience has been that this is the chief difficulty in making a diagnosis. There are some times in the early stages where there seems to be some catarrhal trouble of the smaller bronchi, called typho-pneumonia, which suggests the beginning of pneumonia."

The president appointed Dr. Stockard to give the treatment, and he gave as follows:

"In the treatment, I think that probably the world over, the most usual beginning is calomel, sometimes combined with ipecac; and in this country—that is in the South—it is the custom to give quinine in large doses before the diagnosis is entirely made out. After trying quinine and finding no effect on the fever, the usual course, probably, is to give turpentine emulsion. I think the best plan is to keep down the temperature with water, either by tub or sheet bath, or in some cases where they have an aversion to baths to reduce the temperature by cold enema. I do not favor the administration of the coal-tar derivatives in typhoid fever, as we should harbor the strength of the patient as much as possible, and, therefore, they should not be used to lower the temperature, as it can be accomplished just as well with water and with no bad effect on the patient, but it will have a good tonic effect. The water stimulates the organs to their natural functions and sustains the patient better than any other way. I think the calomel, in the beginning, is important, as it puts the secretions in good condition, and I repeat it whenever necessary. I have never felt any fear in giving a dose, even in the second or third week, but would not give a large dose, simply one-fourth or one-sixth of a grain every few hours will have the effect without any depression and without any amount of peristalsis. Dr. Woodbridge has a treatment consisting of a combination of remedies by which he claims to very much shorten the course of typhoid fever. I saw this last spring and put up a modification of his treatment. Instead of giving small doses at short intervals, I have given large doses at intervals of three or four hours. In view of this discussion to-night, I have jotted down some notes of cases that I have treated this way since last spring.

"A young man, aged 19, was taken three or four days before I saw him and he had a temperature of from 101 to 102. He had taken quinine and was feeling badly; had red tongue and tympanitic condition of the abdomen. I put him on the Woodbridge treatment and a few days after, his condition reached normal.

"The next case was a child five years of age and which I am sure was typhoid fever. When I first saw the case, it had a temperature of 104 and had been sick for some days. That evening, the temperature reached 105 and the tongue was heavily coated and the abdomen distended. I began with the calomel and used the sponge and sheet bath also. The fever gradually declined and also the tympanitic condition of the abdomen, and reached normal condition on the thirteenth day of the disease.

"Child twelve years of age, and I saw first on the 17th of May with a temperature of 102; the tongue was coated and with red edges. I began the treatment, and the next morning, the temperature was 100 and declined rapidly, and the condition was normal on the 23d of May.

"I was called to see a man fifty years of age with a temperature of 103. He had been feeling badly for some time, but worked until the day I was called. His tongue was coated and he had trouble in the abdomen. On the fifth day, his temperature was 99 $\frac{4}{5}$ in the evening.

"Man thirty years of age, who had been feeling badly for a week or more and when I saw him, had a temperature of 103, with considerable uneasiness in the bowels and a rise of fever. I began the treatment on May 20, and the next morning his temperature was 100 $\frac{2}{5}$, on the 23d was 99 $\frac{1}{2}$, and on the 26th was 99.

"On October 16, I was called to see a man who had a perfect case of typhoid fever with a temperature of 101. I put him at once on this treatment and his temperature was normal on the 21st day.

"Case of an actress who had been sick three or four weeks when I was called. She had fever every night and had been taking phenacetine and continuing to work. In the morning, her temperature was 104 and in the evening 105, and had coated tongue, rash, and distended abdomen. On this treatment, the abdominal symptoms subsided and the fever declined steadily.

"Where the poison has spent its force, this treatment will hardly have any effect with the shortening of the course of the disease. In all cases where the temperature reached 102, it was reduced by cold sheet, sponge, or tub bath. I attribute more to the calomel than to the guiacol. I think that to begin with calomel and go through with this treatment, using the baths to lower the temperature, that typhoid fever will be favorably affected by it."

DR. FELDER: I have just passed through an attack of typhoid fever and would say something about it. I want to warn against the use of phenacetine in the third or fourth week, when the temperature gets high and it does not seem to be affected by the baths. My temperature would run up to 105 and 106, and I would take one grain of phenacetine. This would cause a profuse perspiration and I would have to stay in a cold, clammy condition. I would never give it in such cases, as it is not best. In regard to the mode of giving baths, I would state that I had a patient who was very nervous and the preparations for giving the bath would worry her so as to run her temperature up two or three degrees, and it was necessary to give the bath every two hours, as otherwise her temperature would remain at from 103 to 106. In her case, I would put a pint of alcohol in the volume of water with cracked ice, and would wet towels and wrap her body with these wet towels. I would let the towels remain for half an hour until they got hot and the temperature would go down. I kept this up for two weeks and at the same time paid most attention to the nourishment. She could not take sweet milk, and I gave her buttermilk every two hours, also peptones and brandy. These baths were my treatment, except in the latter part of the disease, her pulse became weak and quick, and I gave digitalis to keep up the strength. I also gave turpentine emulsion. She retained her strength remarkably well in such a marked case of typhoid fever. The first day her temperature was 106. She had been in Mobile in the summer, and I suspected malarial fever, and gave quinine and phenacetine, and the next day her temperature went down to normal and below.

I am sure that typhomalarial fever exists, as my attack was of this type. Also, in my case, quinine was beneficial, as the temperature would go up a degree or so, when it was not used. Quinine is the thing in the malarial element.

DR. LOVE: We are all familiar with the symptoms of typhoid fever. When consulted by patients in the first stages, they complain of a dull, languid feeling in the morning and more or less languid in the day; and as the disease progresses, the temperature will rise and usually get high in the afternoon, and tympanitic symptoms will appear, and soreness across the abdomen and in the iliac region. My experience has been that we meet with constipation more than with diarrhœa. In some cases of typhoid fever, you have nervous symptoms and delirium, and one peculiarity of this form of the disease is that the party afflicted, if an adult, almost always follows out his usual occupation. One case was a blacksmith and, when delirious, he would hammer from morning till night, calling to his attendant to turn the iron and telling him when to strike. These usually have diarrhœa and passage of blood from the bowels. When called to a case, I prescribe mild chloride in small doses and often repeat, and then continue the treatment with minute doses of phenacetine, bisulphate of quinine and salol, and after the patient has taken to bed and the temperature rises, I leave off the quinine, specially if the nervous form develops. I want to say here that I have treated in my whole practice in Atlanta but few cases of typhoid fever that did not have complications of malarial fever. I follow the above treatment with turpentine emulsion, and when the fever gets high, I use tincture of digitalis. In tympanitic conditions, I use the turpentine douche, and I give milk and liquid beef as supportive treatment. To act on the bowels, I employ castor oil in preference to calomel. It acts better and softens the fæces. Where there is any tendency to diarrhœa, I use opium, salol, and saccharated bismuth. I use the opium gradually in typhoid fever, as too free use leads to brain complications. In pneumonic complications, I treat as simple pneumonia. For reducing the temperature, there is nothing better than the pack, using wet sheets. All through the case, keep up the supportive treatment, and treat all complications as you would the regular disease.

DR. DUNCAN: I am like Dr. Felder about phenacetine in the last stages of typhoid fever. When you do not know what is the matter and not positive about the fever, it might be given in the first week. The bowel symptoms in typhoid fever vary; in one case you will have constipation, and in another diarrhœa. For constipation in the first week or eight days, I use as enema one drachm of boric acid and ten grains of sal soda in a gallon of water. For tympanitic condition, give three grains of salol and two grains of sulphide of sodium every few hours. These will control most cases. I am in favor of giving calomel in the early stages. To support the heart, I give veratrum, say two drops of veratrum, three drops of digitalis and one drop of oil of cinnamon every three hours. In many cases, the heart wears itself out. This regulates the temperature and improves the heart's action, the patient rests better and the secretions improve. I always give small doses of deodorized tincture of iodine. I am in favor of giving the patients as much buttermilk as they want. It is better than sweet milk, as the sweet milk often forms a coagulum in the stomach. I do not give the turpentine emulsion. I use the turpentine douche in the abdomen, but I use salol and sulphide of soda to control the tympanitis.

Liquid peptones are good. I leave off the solid food and use fluid diet, and tell the patient not to eat solid food for three or four days after the fever has disappeared entirely. The delirium is benefited by fluid extract of gelsemium, or bromide of sodium. Until I came to Atlanta, hæmorrhages from the intestines never gave me any trouble. I had a number of patients who had hæmorrhages from the bowels and they recovered, but when I came to Atlanta, they were different. I have seen a few cases that recovered here in Atlanta that had profuse hæmorrhages. I remember a young man who came from Florida and had typhomalarial fever. I assisted Dr. Stephens in that case. The young man had three profuse hæmorrhages and recovered, but, as a rule, they die here in Atlanta. I have tried various things. I have tried injections of acetate of lead, large doses of tincture of iron, bismuth, also large doses of charcoal. My success with hæmorrhages in Atlanta has not been good."

DR. W. F. WESTMORELAND: Typhoid fever always impressed me that it was a specific trouble and my plan was always the minute we recognized that we had typhoid fever, to recognize the fact that we had, necessarily, more or less ulceration of the intestines, and necessarily, we had a disease that, as far as we know, runs its course. There is no plan of treatment that we know of that has any effect upon it, and we have a patient that has a fight before him, and the best thing is to get him ready for the combat. In dealing with typhoid fever, antipyretics are depressing. All these remedies should be let alone. None can be used with advantage, and they give your patient a less chance, when it comes to the crisis. The cold water, I think, is the best remedy that you can use, with one exception. Frequently you will have a patient that will not stand the cold water. The nervous irritation gives a worse effect. With these patients you can use hot water, or some other method. These means of reducing the temperature are the best I know of. After the patient has passed on until over the course of the fever, and gets into the irritable condition, when they have a cold perspiration from causes which we commonly assign, my plan is to give atropine in full doses, and repeat in full doses three times a day. The skin does not perspire so freely. I never use medicine by the stomach as far as I can possibly avoid it. I use strychnine three or four times a day in full doses, the same as in surgical operations, and am guided by the effect. The remedies should be used entirely for the effect, and simply in that way. No case of typhoid fever is a rule for the fever. When you see them, not only see the fever, but recognize the temperament of the patient. I think sometimes that the thermometer is the worst thing we could have, as it causes us to pay more attention to the temperature than to the treatment of the general condition of the patient.

DR. HURT: I think that typhoid fever is a product of germ or bacteria, and the sooner we recognize the case and make an attack on the conditions the more apt are we to control the fever, and to hold up the strength of the patient, if the fever should be of long duration. It is not hard to arrive at a diagnosis in the regular forms, but in some cases, the symptoms are so masked as to make it difficult. For instance, the patient has the low morning temperature and high evening temperature, without any intermission, and it will run on this way for eight days, when we give mercury and quinine, and on the eighth day find the patient with a normal temperature and seemingly getting well. We then wait twelve hours and discover a rise of temperature, and have to wait from four to eight weeks to get rid of the fever. I believe that to make an attack on typhoid fever, you must recognize the dry, brown tongue, with red edges, headache or constipation, then begin with a good dose of mercury to arouse the secretions and to get the bowels empty. You have then placed the patient where you will take up the line of treatment which will meet the full requirement. If, in some cases, after giving the mercury, we fail to get any moisture on the tongue, alterative doses should be repeated. I have found the patient in condition for the use of salol and phenacetine, but in the early stages of the fever these do not bring the fever down, unless in large doses. My use of these is limited to small doses. I believe it is not safe to give large doses in any case. I believe that two grains will control better than five grains, and five grains will reduce below normal, then the nervous rigor comes on and the temperature rises again. I believe that salol has sufficient antipyretic effect in these instances combined with phenacetine. When placed in the bowels two to four hours, we are able to keep down fermentation and gaseous condition of the bowels. We control fever by keeping the causes inert. As the patient advances to the second, third or fourth week, we have to be careful in the use of any antipyretic. I am inclined to believe that the use of aconite, in small doses, combined with alcohol, is an excellent thing. As to those cases in which delirium occurs, I think it is highly important to pay special attention to the nervous system, and to do that, we have the different nervous sedatives. I use bromide of potash and extract of cannabis indica. While the latter tends to hallucination, yet, it also has a hypnotic tendency. As to hæmorrhage, I have had the misfortune to see ten or twelve cases of violent hæmorrhages from the bowels. My experience has extended in other communities, like Dr. Duncan's, and several have died. This need of restraining hæmorrhage from ulcerated bowels, demands all of a man's tact and energy, and with all that, he is liable to make a sad failure. One case, a boy 15 years of age, bled profusely in the fifth week of the fever, and I used Monsel's solution in glass of water and forced up the rectum. It did good as he had no further movement from his bowels, but on the fifth day after this injection, he passed a blood clot about six inches long. I thought the pressure of the clot arrested the hæmorrhage. He got well. Another case had a hæmorrhage on the fifteenth day and died in a few hours. I saw a negro boy about 18 years of age, and his first symptom which commanded attention was hæmorrhage. He had been feeling badly for a day or two, and at night had half a dozen actions, and the next morning a pool of blood was found at each place where he had an action. I used lead and opium, which restrained bleeding for three days, when his mother gave him castor oil and he bled to death. I think stimulants in typhoid fever are often too early begun. So long as the patient can take nourishment and keeps up, I do not think you should begin stimulating, but should hold this in reserve.

On motion of Dr. Westmoreland, it was decided that the next meeting be devoted to the discussion of what course should be taken in regard to the A Medican ci meral Association, and that a notice be inserted in the papers, inviting all practitioners in the city to be present.

The president appointed Drs. Grandy, Hubbard, and Stockard to get up resolutions on the death of Dr. Powell and report at the next meeting.

W. L. CHAMPION, M.D., Secretary.

---

THINGS WE SHOULD REMEMBER IN THE APPLICATION OF THE FORCEPS.—(Elmer Sothoron, *Virginia Medical Monthly*, Nov., 1895.) The writer relates a case in his own practice in which the mother refused the aid of the forceps while the child's vertex rested against the symphysis for three hours. At the end of this time she consented to their use, and the doctor delivered a dead baby. The moral is self-evident. Nevertheless, he thinks we should never attempt to force nature by the use of the forceps unless the life of mother or child is in danger. We should remember that forceps act as a compressor as well as a tractor, and the perils arising from this fact. The physician should diagnose accurately the position of the fœtal head, and know the exact course which it has to pursue in its exit, in order that undue or wrongly directed force be not employed in the instrumental extraction. We should never attempt to apply forceps without a proper anæsthetic. Assistants to hold the knees firmly are of great service in preventing rupture of the perineum. Plenty of time must be taken to apply the forceps properly, in order that they do not slip or crush the child's head. We should always guide with a finger each blade to its safe and proper place, being especially careful not to catch the thin and stretched os uteri between the forceps and the presenting part. We should always give a vaginal injection, empty the bowels and bladder and cleanse the forceps before applying them. The time for their application is to be determined by our own knowledge and common sense, and not by the entreaties of the patient or her friends.—*Denver Medical Times.*

